# Characteristics of Hospital Workers Using a Wellbeing Center Implemented During the COVID-19 Pandemic to Prevent the Emotional Impacts of the Crisis

**DOI:** 10.3389/fpubh.2022.913126

**Published:** 2022-07-04

**Authors:** Marguerite d'Ussel, Frédéric Adam, Audrey Fels, Gilles Chatellier, François Philippart

**Affiliations:** ^1^Chronic Pain Unit, Groupe Hospitalier Paris Saint-Joseph, Paris, France; ^2^Department of Anesthesia, Groupe Hospitalier Paris Saint-Joseph, Paris, France; ^3^Clinical Research Department, Groupe Hospitalier Paris Saint-Joseph, Paris, France; ^4^Université Paris Cité, Paris, France; ^5^Department of Intensive Care Medicine and Reanimation, Groupe Hospitalier Paris Saint-Joseph, Paris, France

**Keywords:** anxiety, burnout, COVID-19, depression, healthcare workers, post-traumatic stress disorder, mental health, wellbeing center

## Abstract

**Introduction:**

The COVID-19 pandemic has posed an unprecedented challenge worldwide for healthcare workers (HCWs) and other hospital employees. Disruptions in work and personal life may have led to mental health problems. To prevent or limit the severity of such issues, a local initiative has been implemented in a French hospital: a dedicated lounge, also called “*Bulle*” (literally bubble and meaning safe space) has been created to provide a quiet caring environment and health support. Other similar wellbeing centers have been implemented in other countries, but very little data are available on their practical effectiveness. The purpose of our study was to assess what type of hospital workers have frequented the *Bulle* and to describe their psychological state in terms of anxiety, depression, and post-traumatic stress disorder (PTSD) just after the first wave, compared to those who had not come to the *Bulle*.

**Methods:**

From 15 July to 1 October 2020, a cross-sectional survey was conducted among all workers, collecting demographic information, professional data (experience and satisfaction), emotional experience during the first wave of COVID-19, and psychological specificities, including a history of burnout or symptoms of anxiety, depression, and PTSD. We asked them if they had accessed the *Bulle* or not.

**Results:**

A total of 675 employees (out of 2,408; 28.0%) fully completed the survey. Approximately 199 respondents (29%) reported having accessed the *Bulle* during the first wave of the pandemic. Significant symptoms of anxiety, depression, and PTSD were reported by, respectively, 41, 20, and 14% of the participants. Logistic regression analysis showed no relationship between the use of the *Bulle* and the prevalence of later psychological symptoms. However, employees who benefit from the solicitation of the psychological support team in their hospital unit were secondarily more prone to come to the *Bulle* [odds ratio (OR), 2.24; 95% confidence interval (95% CI): 1.09; 4.59].

**Conclusion:**

Anxiety, depression, and PTSD were common after the first part of the COVID-19 pandemic, and the attendance in quiet and wellbeing spaces seemed easier with direct internal proactive intervention by psychological teams.

## Introduction

There has been widespread emotional distress caused by the COVID-19 pandemic. Early events associated with this new and highly infectious virus were a source of anxiety for populations worldwide (1–5). This anxiety could have been explained by the fear of an unknown life-threatening viral pneumonia, the absence of treatment, and confusing information from physicians, scientists, and politicians about sanitary measures, the relevance of lockdown, and the risk of professional and social interactions (6–12). All these changes in daily life and people's increased isolation from normal social interaction and family relationships may have had a psychological impact and led to post-traumatic stress disorder (PTSD) (6, 13).

In addition to the stress factors of the general population, frontline healthcare workers (HCWs) had to face many other specific issues. The excessive number of confirmed and suspected cases, the overwhelming workload, the lack of personal protection equipment, the widespread media coverage, the depletion of specific drugs, and the feeling of being inadequately supported all may have contributed to the mental burden of these HCWs. These lifestyle changes significantly impacted their routine, specifically in terms of job responsibility and social isolation, and notably affected their psychological health and stress levels (14–18). This impacted all HCWs: doctors, nurses, and other medical personnel in contact with suspected or confirmed COVID-19 cases, but also pharmacists, hospital support staff, laboratory technicians, admission/ward clerks, and all hospital workers. The latter HCWs may not have been directly involved in providing assessment or treatment, but may have operated in similarly stressful clinical settings, and were at heightened risk of being infected. The pandemic and all its difficult outcomes have led to a higher risk of anxiety, depression, burnout, and more generally emotional distress among hospital staff (7, 11, 16, 17, 19).

The COVID-19 pandemic has generated an increased sense of urgency to mobilize crisis response provisions to protect the wellbeing of hospital workers. Some hospital trusts have engaged teams to rapidly create respite spaces for staff (20, 21), albeit the same concept has been labeled in many ways: “bubble rooms,” “wobble rooms,” “time-out rooms,” “chill-out rooms,” “safe rooms,” “rainbow rooms,” and “wellbeing centers” (14, 22–25). These facilities are usually located in non-COVID areas and, for frontline workers, provide an opportunity for staff to remove themselves from the clinical environment and gain solace from the pressures of dealing with coronavirus. They are intended to provide an optimistic and positive atmosphere to help staff with the impact of the crisis: small enough to be perceived as “homely” spaces but large enough to maintain privacy and appropriate social distancing (22). Depending on the country or the hospitals, they could be set differently, indoors and/or outdoors, with a garden view (23). The lounges could offer music, refreshments (22, 23), various activities, such as relaxing sessions, physiotherapist interventions (23, 24), or even immersive experiences (25), and learning packages (22). Psychological support could also be provided directly by specialists (23) or suggested by “buddies” helping staff (22). In addition to physical lounges, some teams also provided virtual spaces for HCWs, providing regular online group-based support focused on honest expression of feelings and self-care (26).

Despite the large number of spontaneous interventions to improve the resilience of HCWs during this psychologically distressing time, very little data are available about the use of these wellbeing centers (24). A recent review has shown that only a few countries have published specific psychological support intervention protocols for HCWs. Given the heterogeneity of protocols and the clinical outcomes studied, it is also impossible to determine whether one protocol has better outcomes than the others. The authors of the review conclude that further research is needed to find out the best ways to support the resilience and mental wellbeing of HCWs (27).

In our French hospital, we have also implemented a wellbeing center that we call *Bulle* (literally bubble, meaning safe space). The purpose of our study was to assess what type of hospital workers have frequented the *Bulle* and to describe their psychological state in terms of anxiety, depression, and PTSD, just after the first wave, compared to those who had not come to the *Bulle*. This study can help us to improve our *Bulle* to attract the most fragile hospital staff and mitigate the psychological impact of the COVID-19 crisis or other difficulties on each of them.

## Context

### Hospital Structure

The Groupe Hospitalier Saint-Joseph (GHSJ) is a two-site hospital organization (Saint-Joseph and Marie Lannelongue) located in the Great Paris area. During the first wave of the COVID-19 pandemic (between 1 March and 29 May 2020) 1,177 patients had been seen *via* the emergency ward, 834 were hospitalized, 132 needed intensive care, and 100 died.

### Our *Bulle* Concept

We implemented the *Bulle* in the early stage of the first wave on 27 March 2020 to support the resilience and mental health of frontline workers by providing targeted and individualized interventions in an adapted environment. The *Bulle* was designed to favor relaxation and coping against stress, to strengthen camaraderie and support and also to provide professional healthcare. The concept was born out of a medical team working to support self-management in people with chronic pain. This multidisciplinary team served to propose an integrative approach for these patients, to improve their quality of life, and alleviate the emotional difficulties related to their pain. This medical approach is currently recognized in the field of chronic diseases (28, 29), and in particular in oncological support care (28–30). It quickly appeared logical to replicate this approach to the care of the medical and non-medical staff, especially as the healthcare professionals of the team could no longer receive their patients in the hospital, which was only dedicated to COVID-19 infected or emergency patients. To encourage as many staff as possible to come and take care of themselves, we designed the *Bulle* as a place of resources, quietness, and friendship, as it had been implemented in Toronto during the SARS outbreak (31). Open with free access to all hospital workers from 11.30 am to 8 pm, every day, including public holidays except on weekends, our lounge was managed by health professionals working in our institution. We built the *Bulle* as a soothing space (comfortable seating, relaxing music, low-level lighting, plants, an aromatherapy pod, etc.), welcoming and friendly with hot or cold drinks and regressive sweets. From the outset, the *Bulle* has let available self-massage chairs, musical relaxation sessions with the “Music Care” digital tablets (32), micro-nap areas, or creative workshops. Online appointments were also opened for physiotherapy, osteopathic manual therapy to treat or limit the occurrence of musculoskeletal disorders, individual and group sessions of relaxation therapy and hypnosis led by volunteer practitioners from our hospital. We quickly enriched the therapeutic offer by opening consultations with a psychologist, as well as with an anti-smoking doctor.

### The Survey

To evaluate the relevance, congruence with hospital employees' expectations, and potential appropriateness of our *Bulle*, we designed a survey concerning the whole GHSJ staff, i.e., all those who had worked during the first wave. The questionnaire was sent 3 months after the first peak of the pandemic, between 15 July and 1 October.

We used a “Microsoft Forms” online questionnaire due to compliance with French data protection laws and the potential for access control, encryption, and account security. Potential respondents were solicited using the hospital mailing list. A specific link was also made available on the intranet network of the group. At last, a QR code displayed on the screensavers of all computers allowed people to get the survey information and answer it at any moment. The survey included five components that were identified from a literature review (33–36) and a local Delphi method. They were: (the submitted questionnaire is accessible as supporting information—Supplementary Appendix S1).

1. Personal information (demographics, exposure to COVID-19, and history of burnout).
2. Professional experience and fears at the time of the survey.
3. Emotional experience during the first wave, and degree of job satisfaction:
  - Did the COVID-19 crisis make you anxious? If so: for yourself, your family, others, or your work?
  - Were you afraid of infecting your loved ones?
  - Do you usually feel good at work?
  - Do you currently feel good at work?
4. The Hospital Anxiety and Depression Scale (HADS). The HADS is a 14-item auto questionnaire that includes seven items on symptoms of anxiety, and seven items on symptoms of depression (37). A cutoff score >7 was used for each subscale for detecting significant symptoms of anxiety or depression (38, 39). Its French translation has been validated among many populations (40–42).
5. The PTSD Checklist (PCL). The PCL is a 17-item self-report measure reflecting The Diagnostic and Statistical Manual of Mental Disorders-IV (DSM-IV) symptoms of PTSD (43). A cutoff score >44 was used for detecting significant symptoms of PTSD (44). It was made clear in the questionnaire that the stressful event to which respondents were referring was the health crisis [considered to have been over a month old at the time of the survey to consider the symptoms as belonging to a PTSD and not an acute stress state (45)].

From the results of this survey, we extracted data on the staff who had accessed the *Bulle* (those who came more than two times a month).

The protocol has been performed by following the Declaration of Helsinki and approved by the Groupe Ethique et Recherche Médicale/ Ethics and Medical Research Group (GERM) from the Hospital Paris Saint-Joseph, institutional ethics committee (IRB No. 00012157).

According to French regulations (46) no written informed consent was required and an information note setting out the purpose of the research is sufficient for his category of study.

The authors guarantee the anonymity of all collected data.

### Statistical Analysis

All results are reported as numbers (percentage). Variables were compared using either the χ^2^ test or Fisher's exact test, as appropriate.

The relationship of the characteristics of workers who frequented with attendance or not to the *Bulle* was assessed using logistic regression. Odds ratios (ORs) and their 95% confidence intervals (95%CIs) were calculated using univariable and multivariable logistic regression models. For the multivariable analysis, variables of interest were selected according to their statistical significance in the univariable analysis (critical *p*-value for entry into the model: 0.2). Considering the absence of the *Bulle* at the Hospital Marie Lannelongue location, we excluded Marie Lannelongue workers before analysis.

The analysis was performed with the R software (the R project for statistical computing, https://www.r-project.org/). All tests were two-sided, and the value of *p* < 0.05 was considered statistically significant.

## Results

### General Responding Population

The survey was fully completed by 675 of the 2,408 (28.0%) employees working at the HPSJ (Table 1). Most participants were aged from 26 to 50 years, and 82.1% were women. The majority (433; 66.8%) lived with a partner, and 339 (84.8%) had at least one child. Among the respondents, 199 (29.5%) reported that they have accessed the *Bulle* during the first wave of the pandemic.

**Table 1:** Sociodemographic and emotional characteristics related to COVID-19 and job satisfaction information according to access to the *Bulle*, among Paris Saint-Joseph Hospital employees.

	**Total (*n* = 675)**	***Bulle*** **no (*n* = 476)**	***Bulle*** **yes (*n* = 199)**	**OR [95% CI]**	**P**
**Gender**					
Men	121 (17.9%)	88 (18.5%)	33 (16.6%)	1.00	0.56
Women	554 (82.1%)	388 (81.5%)	166 (83.4%)	1.14 [0.74; 1.77]	
**Age**					
18–25	58 (8.6%)	30 (6.3%)	28 (14.1%)	1.00	<0.001
26–33	161 (23.8%)	101 (21.2%)	60 (30.2%)	0.64 [0.35; 1.17]	
34–41	144 (21.3%)	108 (22.7%)	36 (18.0%)	0.36 [0.19; 0.68]	
42–50	112 (16.6%)	90 (18.9%)	22 (11.1%)	0.26 [0.13; 0.52]	
50–60	162 (24.0%)	121 (25.4%)	41 (20.6%)	0.36 [0.19; 0.68]	
+60	38 (5.6%)	26 (5.5%)	12 (6.0%)	0.49 [0.21; 1.16]	
**Marital situation (*****N*** **= 648)**					
Single	215 (33.2%)	145 (32.1%)	70 (35.7%)	1.00	0.37
In couple	433 (66.8%)	307 (67.9%)	126 (64.3%)	0.85 [0.6; 1.21]	
**Familial situation (*****N*** **= 400)**					
No child	61 (15.3%)	39 (13.0%)	22 (21.8%)	1.00	0.035
One or several children	339 (84.7%)	260 (87.0%)	79 (78.2%)	0.54 [0.3; 0.96]	
**Profession**					
Administrative healthcare	124 (18.4%)	94 (19.8%)	30 (15.1%)	1.00	0.09
Medical professional	112 (16.6%)	78 (16.4%)	34 (17.1%)	1.37 [0.77; 2.43]	
Caregiver	315 (46.7%)	216 (45.4%)	99 (49.7%)	1.44 [0.89; 2.31]	
Other caregiver	57 (8.4%)	38 (8.0%)	19 (9.6%)	1.57 [0.79; 3.11]	
Midwife	21 (3.1%)	20 (4.2%)	1 (0.5%)	0.16 [0.02; 1.22]	
Others	46 (6.8%)	30 (6.3%)	16 (8.0%)	1.67 [0.8; 3.48]	
**Professional experience in the same hospital unit**					
<5 years	377 (55.8%)	245 (51.5%)	132 (66.3%)	1.00	<0.001
>5 years	298 (44.2%)	231 (48.5%)	67 (33.7%)	0.54 [0.38; 0.76]	
***Professional experience***					
<5 years	163 (24.1%)	96 (20.2%)	67 (33.7%)	1.00	<0.001
>5 years	512 (75.9%)	380 (79.8%)	132 (66.3%)	0.5 [0.34; 0.72]	
**Duty station during first wave**					
***COVID-19 –Unit assignment***					
No	350 (51.8%)	257 (54.0%)	93 (46.7%)	1.00	0.09
Yes	325 (48.2%)	219 (46.0%)	106 (53.3%)	1.34 [0.96; 1.86]	
***No COVID-19 –Unit assignment***					
No	374 (55.4%)	268 (56.3%)	106 (53.3%)	1.00	0.47
Yes	301 (44.6%)	208 (43.7%)	93 (46.7%)	1.13 [0.81; 1.58]	
***Remote work***					
No	583 (86.4%)	396 (83.2%)	187 (94.0%)	1.00	<0.001
Yes	92 (13.6%)	80 (16.8%)	12 (6.0%)	0.32 [0.17; 0.6]	
***No-clinical professional activity***					
No	556 (82.4%)	386 (81.1%)	170 (85.4%)	1.00	0.18
Yes	119 (17.6%)	90 (18.9%)	29 (14.6%)	0.73 [0.46; 1.15]	
**COVID-19 patients management**					
Never/rarely	368 (54.5%)	272 (57.1%)	96 (48.2%)	1.00	0.034
Regularly/frequently	307 (45.5%)	204 (42.9%)	103 (51.8%)	1.43 [1.03; 1.99]	
**Respondents who were infected or having infected colleagues or relatives**					
No	258 (38.2%)	171 (35.9%)	87 (43.7%)	1.00	0.06
Yes	417 (61.8%)	305 (64.1%)	112 (56.3%)	0.72 [0.52; 1.01]	
**Current job satisfaction**					
No	166 (24.6%)	118 (24.8%)	48 (24.1%)	1.00	0.85
Yes	509 (75.4%)	358 (75.2%)	151 (75.9%)	1.04 [0.71; 1.52]	
**History of professional burnout or depression**					
No	528 (78.2%)	376 (79.0%)	152 (76.4%)	1.00	0.45
Yes	147 (21.8%)	100 (21.0%)	47 (23.6%)	1.16 [0.78; 1.72]	
***Date of professional burnout or depression history (n =147)***					
−3 years	57 (38.8%)	42 (42.0%)	15 (31.9%)	1.00	0.24
+3 years	90 (61.2%)	58 (58.0%)	32 (68.1%)	1.54 [0.74; 3.21]	
**Anxiety during COVID-19 first wave**					
No	256 (37.9%)	186 (39.1%)	70 (35.2%)	1.00	0.34
Yes	419 (62.1%)	290 (60.9%)	129 (64.8%)	1.18 [0.84;1.67]	
***Anxiety for oneself (n = 419**)*					
No	181 (43.2%)	132 (45.5%)	49 (38.0%)	1.00	0.15
Yes	238 (56.8%)	158 (54.5%)	80 (62.0%)	1.36 [0.89; 2.08]	
***Anxiety for family (n = 419)***					
No	57 (13.6%)	41 (14.1%)	16 (12.4%)	1.00	0.63
Yes	362 (86.4%)	249 (85.9%)	113 (87.6%)	1.16 [0.63; 2.16]	
***Anxiety for others (n = 419)***					
No	241 (57.5%)	167 (57.6%)	74 (57.4%)	1.00	0.97
Yes	178 (42.5%)	123 (42.4%)	55 (42.6%)	1.01 [0.66; 1.54]	
***Anxiety at work*** (n = 419)					
No	207 (49.4%)	144 (49.7%)	63 (48.8%)	1.00	0.88
Yes	212 (50.6%)	146 (50.3%)	66 (51.2%)	1.03 [0.68; 1.56]	
**Fear of contaminating relatives**					
No	171 (25.3%)	121 (25.4%)	50 (25.1%)	1.00	0.94
Yes	504 (74.7%)	355 (74.6%)	149 (74.7%)	1.02 [0.69; 1.49]	
***Between March 15**^**th**^ **and May 15**^**th**^**, did you meet with the psychological support team in your unit?***					
No	592 (87.7%)	436 (91.6%)	156 (78.4%)	1.00	<0.001
Yes	83 (12.3%)	40 (8.4%)	43 (21.6%)	3 [1.88; 4.8]	
***Do you think this passage of the support cell has been beneficial on your emotional level? (n = 83)***					
Not at all / not very beneficial	15 (18.1%)	13 (32.5%)	2 (4.6%)	1.00	0.002
Quite beneficial	36 (43.4%)	17 (42.5%)	19 (44.2%)	7.26 [1.43; 36.94]	
Very beneficial	32 (38.5%)	10 (25.0%)	22 (51.2%)	14.3 [2.7; 75.65]	
**Usual job satisfaction**					
Never/sometimes	61 (9.0%)	40 (8.4%)	21 (10.5%)	1.00	0.37
Often/Always	614 (91.0%)	436 (91.6%)	178 (89.5%)	0.78 [0.45; 1.36]	
Anxiety symptoms					
**(HADa > 7)**					
No	399 (59.1%)	286 (60.1%)	113 (56.8%)	1.00	0.43
Yes	276 (40.9%)	190 (39.9%)	86 (43.2%)	1.15 [0.82; 1.6]	
Mean HADa (SD)	7.04 (±4.07)	6.81 (±4.07)	7.50 (±4.02)		0.093
**Depression symptoms**					
**(HADd > 7)**					
No	540 (80.0%)	384 (80.7%)	156 (78.4%)	1.00	0.50
Yes	135 (20.0%)	92 (19.3%)	43 (21.6%)	1.15 [0.77; 1.73]	
**Mean HADd (SD)**	4.30 (±3.74)	4.17 (±3.74)	4.43 (±3.69)		0.29
PTSD					
**(PCL > 44)**					
No	581 (86.1%)	413 (86.8%)	168 (84.4%)	1.00	0.42
Yes	94 (13.9%)	63 (13.2%)	31 (15.6%)	1.21 [0.76; 1.93]	
**Mean PCL (SD)**	28.9 (±12.8)	28.3 (±12.6)	30.6 (±13.4)		0.081

*OR, odds ratio; CI, confidence interval; SD, standard deviation*.

Most respondents were caregiving staff (*n* = 315; 46.7%) (nurses, assistant nurses, and nurse managers); 124 participants (18.4%) were health administrative workers (secretary, logistic manager, and pharmaceutical assistant), 112 (16.6%) were doctors or pharmacists, 57 (8.4%) were other caregivers (physiotherapist, stretcher-bearer, radiologic technologist, and psychologist), 21 (3.1%) were midwives, and 46 (6.8%) had other occupation. The length of professional experience was more than 5 years for 512 participants (75.9%) and 377 (55.9%) were in the same workplace for <5 years. With regard to the work location, 325 respondents (48.2%) reported working in a “COVID-19 Unit” (ward for patients with COVID-19) during the wave, 301 (44.6%) in another clinical unit, 92 (13.6%) worked at home, and 119 (17.6%) worked as non-clinical support places. Among the frontline HCWs, 307 (45.5%) reported having taken care of patients with COVID-19 frequently (every working day) or regularly (at least 1 day a week).

In addition to their shifts, 417 (61.8%) reported that they themselves, a colleague, a friend, or a close relative had been infected with SARS-CoV-2.

### Psychological Issues in the Responding Population

Most employees reported a high level of job satisfaction (*n* = 509; 75.4%). A total of 147 participants (21.8%) reported a previous history of burnout or depression. Most of the participants spoke of overwhelming levels of worry and concern related to COVID-19 first wave (*n* = 419; 62.1%), especially associated with their family (*n* = 362, 86.4%), themselves (*n* = 238; 56.8%), or their working conditions (*n* = 212; 50.6%). Almost three-quarters of the participants reported being afraid of infecting their relatives (*n* = 504; 74.7%).

Clinically, significant levels of symptoms of anxiety (according to the HAD A scale > 7 points), depression (according to the HAD D scale > 7 points), and PTSD (according to the PCL scale > 44 points) were found, respectively, in 276 (40.9%), 135 (20.0%), and 94 (13.9%) respondents.

Notably, age was not associated with higher measures of anxiety, depression, or PTSD. Staff younger than 41 years had no more symptoms of anxiety (56.6% vs. 53.4%, *p* = 0.45), depression (52.5% vs. 47.5%, *p* = 0.47), or PTSD (60.4% vs. 39.6%, *p* = 0.22) than older ones.

### Characteristics of Employees Who Had Accessed the *Bulle*

Some characteristics were compared between those who had accessed the *Bulle* and those who had not. As shown in Table 1, participants who had accessed the *Bulle* during the first wave were younger, aged 18 and 25 years (14.1 vs. 6.3% *p* < 0.001). They had shorter professional experience (33.7 vs. 20.2%, *p* < 0.001) and had been in the same hospital location for less time (66.3 vs. 51.5%, *p* < 0.001). The *Bulle* was most likely to be accessed by staff without children than among those with children (21.8 vs. 13.0%, *p* = 0.035), and among those who worked with patients with COVID-19 than among those who did not (51.8 vs. 42.9%, *p* = 0.034). Hospital employees who met the psychological support team in their working unit were more likely to access the *Bulle* than those who did not (21.6 vs. 8.4%, *p* < 0.001), and more often perceived that this visit had a large emotional benefit (51.2 vs. 25.0%, *p* = 0.002).

In the multivariable analysis (Table 2), few independent characteristics were found to be statistically different between employees who came at the *Bulle* and others. Those who met the psychological team in their hospital unit were more prone to come to the *Bulle* than those who did not (OR, 2.2; 95% CI: 1.1–4.6: *p* = 0.027). Hospital workers who were remote workers came to the *Bulle* less often than others (OR 0.10; 95% CI 0.02–0.31; *p* < 0.001).

**Table 2:** Multivariable logistic regression analysis of characteristics of employees associated with access to the *Bulle*.

	**OR [95% CI]**	**p**
**Age**		
26–33 (vs. 18–25 years)	0.95 [0.22; 4.28]	0.95
34–41 (vs. 18–25 years)	0.42 [0.08; 2.19]	0.30
42–50 (vs. 18–25 years)	0.35 [0.06; 1.96]	0.23
50–60 (vs. 18–25 years)	0.52 [0.10; 2.85]	0.44
+ de 60 (vs. 18–25 years)	0.76 [0.12; 4.95]	0.77
Familial situation one or several children (vs. no child)	0.54 [0.25; 1.18]	0.11
**Profession**		
Medical professional (vs. administrative healthcare)	0.76 [0.33; 1.76]	0.53
Caregiver (vs. administrative healthcare)	0.74 [0.34; 1.61]	0.44
Other caregiver (vs. administrative healthcare)	0.41 [0.10; 1.41]	0.18
Midwife (vs. administrative healthcare)	0.11 [0.01; 0.71]	0.05
Others (vs. administrative healthcare)	1.09 [0.31; 3.51]	0.89
**Professional experience in the same hospital service >5 years (vs**. ** <5 years)**	0.76 [0.42; 1.36]	0.35
**Professional experience >5 years (vs**. ** <5 years)**	1.62 [0.69; 4.00]	0.28
**COVID-19 –unit assignment yes (vs. No)**	0.52 [0.25;1.02]	0.06
**Remote work yes (vs. no)**	0.10 [0.02; 0.31]	<0.001
**No-clinical professional activity yes (vs. no)**	0.91 [0.45; 1.80]	0.79
**COVID-19 patients management regularly/frequently (vs. never/rarely)**	1.34 [0.69; 2.62]	0.38
**Respondents who were infected or having infected colleagues or relatives yes (vs. no)**	0.69 [0.41; 1.15]	0.16
**Anxiety during COVID-19 first wave**		
Anxiety for oneself (vs. no anxiety)	1.78 [1.00; 3.22]	0.05
Anxiety for family, for others or at work (vs. no anxiety)	1.05 [0.55; 1.99]	0.89
**Between March 15 and May 15, did you meet with the psychological support team in your unit? yes (vs. no)**	2.24 [1.09; 4.59]	0.027

*OR, odds ratio; CI, confidence interval*.

The importance of exposure to patients with COVID-19 as an independent factor associated with the use of the *Bulle* was searched. Our multivariable analysis found no association (OR 1.34; 95% CI 0.69–2.62; *p* = 0.38).

## Discussion

Our study emphasizes the emotional impact of the COVID-19 pandemic on hospital staff. The employees who completed the questionnaire were mainly HCWs, with more than 5 years of experience in their job. Approximately 50% worked in contact with patients with COVID-19 during the first wave. The perception of having been anxious during the first wave was frequent among HCWs who completed the survey, mainly because of the risk of SARS-CoV2 transmission from the hospital to their relatives. High depression scores were observed in 20% of respondents and 41% for anxiety scores.

Other studies in the literature concerning the HCWs during the COVID-19 pandemic reported a prevalence of anxiety from 23 to 51%, and depression from 22 to 50% (47–49), depending on the scales used. A systematic review of 29 studies reported a median prevalence of 24% for anxiety and 21% for depression among HCWs (50). The prevalence of PTSD symptoms among HCWs reported in the literature varied between 71% (51), 21% (52), and 15% (53), which is more than our rate of 14%.

As previously demonstrated by several groups since the beginning of the COVID-19 pandemic, wellbeing centers seem to be efficient and useful (21–23, 25, 54, 55). The protocols are quite different, in terms of setting (historical cloister and garden to unused laboratory), staff (“wellbeing buddies,” psychiatrists, psychologists, social workers, and physical therapists), tools (quiet space, massages, Pilates, relaxation therapy, sensory rooms, etc.). However, the purpose is always similar: to provide a brief break from the global turmoil of healthcare during a hospital frontline work day (22, 23, 25, 56). Current studies emphasize the needs expressed needs by hospital staff and the testimony of individual benefits of one or the other part of the wobble room, such as quietness and psychological support (22). Notably, although associated with multiple biases, a large part of respondents stated that the use of the *Bulle* has had a positive impact on their emotional status, similar to what has been previously published (22, 56). In the same way, social and administrative workers seem to get benefits from the *Bulle*. Few previous studies were devoted to this part of the hospital staff (16, 23). However, some surveys found, as we did, that non-clinical hospital workers had high risks for depression, insomnia, anxiety (57), and PTSD (58) during a health crisis. As hospital employees, they suffer from double stress due to both community and potential patient exposure in many situations. It is noteworthy that if administrative jobs offer large opportunities for remote work and thus a lower risk of COVID-19 accidental exposure (59), they are exposed to an increased risk of stress factors associated with social isolation (60). Another reason could be that the logistic staff and others working in the hospital might not be as psychologically prepared as doctors and nurses (58). This observation should let wellbeing centers be freely open to any type of profession. Another interesting point is the reason expressed for anxiety, as a retrospective experience. Regardless of the use of the *Bulle*, the major reason for this anxiety was the fear of viral transmission to their relatives, rather than their own risk of disease. This is a major issue, already underlined in general studies on the stress of HCWs during the COVID-19 pandemic (61–63).

A remaining question is how to reach our “target population” of most affected persons? (16). In our work, anxiety seems to remain a few months after the first severe wave of the pandemic. Interestingly, our multivariable analysis highlighted the role of intervention of the support team in the probability of accessing the *Bulle*. This couple of psychologist and medical doctor visited care departments to meet professionals and exchange with them. It was agreed that these couples would promote the *Bulle* to encourage staff to go there. Distressed workers may suffer from psychological inhibition, and the proactive intervention of trained professionals may increase “recruitment” of the most vulnerable workers. In our survey, meeting this psychological support team, before going to the *Bulle*, seems to be associated with increased attendance at the wellbeing center, even if such data should be confirmed in prospective studies. At last, we observed that being married and having children were associated with a lower frequency of the use of the *Bulle*. This point was unexpected. Fear of viral transmission to relatives is associated with increased anxiety and having children would increase anxiety. Considering the possible use of a wellbeing center during working hours, this link seems not to be associated with lack of time, leading us to consider relatives as a protective psychological factor. However, this point is not observed in large studies (16). Nonetheless, small cohorts already showed a potential protective effect of assistance from relatives, families, friends, and even patients (64).

Our work has several limitations. First, it is a retrospective survey on the use of the *Bulle* center, leading to memory biases. However, considering the evaluation of anxiety, depression, and PTSD at the time of the evaluation questionnaire, the responses are probably relevant for evaluating mental status a few months after the first wave of the pandemic. The voluntary nature of participation in the survey may lead to problems of sample bias (65). Second, in this cross-sectional study, we did not randomize the use of the *Bulle*, leading to difficulties to evaluate the effect of the wellbeing center on anxiety, depression, or PTSD. Ethical issues led us not to limit access to our center. Conversely, prospective studies about specific tools are acceptable and we plan to confirm the observed benefit with randomized works in the future. Third, due to the survey response rate, there is a unit non-response error that results from a failure to collect information on all units in the selected sample. Unit non-response is a source of non-sampling error that can influence our survey. Estimates based only on respondents may be biased if the characteristics of respondents and non-respondents are not the same.

The choice of scales used to assess an emotional state has been carefully considered: in France, the HAD scale is the reference scale for assessing anxiety and depression in any type of population because it is easy and quick to complete, and its French translation has been validated (40–42), unlike several other international scales assessing the same symptoms.

On the other hand, we have chosen PCL, which refers to DSM-IV rather than the newer version PCL-5 because of the controversy about the causal link between COVID-19 and PTSD. Indeed, frontline hospital staff in a region particularly affected by the epidemic, who had to reorganize all their work, were confronted with much more numerous serious patients than usual, with the risk of transmitting the virus to their relatives due to the lack of personal protective equipment, etc. have been exposed to a particularly stressful event that may meet the DSM-V definition (66)—direct exposure to the traumatic event (TE) or direct witness (of a TE happening to others), repeated or extreme exposure to aversive situations (e.g., caregivers, rescue workers, and hospital staff). Some authors, agree with that point of view (67, 68). However, PTSD should not be considered a concept that applies to COVID-19 in general (69–72). As DSM-V does not allow to consider COVID-19 as a TE responsible for PTSD, we used, as previously done, the DSM-IV parameters as inclusion criteria (31, 73, 74).

Our study has several strengths. This is a large study, which makes the result powerful. The sample studied is homogeneous: all respondents work in the same university hospital in Greater Paris, which was severely affected by the first wave of the pandemic. The distribution of the study population is representative of the total hospital population and includes all types of professions, including administrative staff. The relevance of the implementation of this *Bulle* innovation by an experienced clinician has been confirmed by the deployment of similar lounges in several other French hospitals subsequently.

It is now necessary to carry out studies to highlight its usefulness and measure its impact on the mental health of hospital staff. We want, for example, to evaluate the impact of the use of *Bulle* on the level of anxiety, heart rate variability (HRV), and the quality of life over 3 and 6 months.

## Conclusion

During the COVID-19 pandemic, a number of wellbeing centers were set up in hospitals around the world. This was an early intervention to mitigate the psychological impact of the COVID-19 crisis on HCWs. Although we cannot recommend one type of intervention over another because no study has been conducted to show their benefits on the hospital workers, high-quality rest spaces should be essential for staff wellbeing. Our survey highlights that wellbeing centers are frequented by staff from all occupational groups and might be promoted equally among them. Potential recruitment of frail workers by specific intervention teams may be relevant. The demonstration of the benefits of these spaces needs to be provided by specific prospective studies.

## Data Availability Statement

The original contributions presented in the study are included in the article/Supplementary Material, further inquiries can be directed to the corresponding author.

## Ethics Statement

The studies involving human participants were reviewed and approved by Institutional Ethics Committee (IRB number 00012157): the GERM (Groupe Ethique et Recherche Médicale/ Ethics and Medical Research Group) from the Hospital Paris Saint-Joseph. Written informed consent for participation was not required for this study in accordance with the national legislation and the institutional requirements.

## Author Contributions

MU, FA, and FP: data curation, investigation, project administration, visualization, draft writing, and final version correction. MU, FA, FP, AF, and GC: conceptualization, formal analysis, methodology, and validation. AF and GC: review and editing. All authors contributed to the article and approved the submitted version.

## Conflict of Interest

The authors declare that the research was conducted in the absence of any commercial or financial relationships that could be construed as a potential conflict of interest.

## Publisher's Note

All claims expressed in this article are solely those of the authors and do not necessarily represent those of their affiliated organizations, or those of the publisher, the editors and the reviewers. Any product that may be evaluated in this article, or claim that may be made by its manufacturer, is not guaranteed or endorsed by the publisher.
