# Supplementary material for: Characteristics of Hospital Workers Using a Wellbeing Center Implemented During the COVID-19 Pandemic to Prevent the Emotional Impacts of the Crisis
Source: Front Public Health. 2022 Jul 4;10:913126. doi: 10.3389/fpubh.2022.913126 (PMC9289445; doi:10.3389/fpubh.2022.913126)

**S1 Appendix: submitted questionnaire for the survey**

1. What is the place of your professional practice

- Hospital Paris Saint-Joseph
- Hospital Marie-Lannelongue

1. Did you work between March 15^th^ and May 15th?

- Yes
- No

If no : end of the questions.

1. Are you

- A woman
- A man

1. How old are you ?

- 18-25 ans
- 26-33 years
- 34-41 years
- 42-50 years
- 50-60 years
- + 60 years

1. What is your profession ?

- Caregiver (nurse, assistant nurse, nurse manager)
- Administrative healthcare
- Medical professional (physician, pharmacologist, biologist)
- Other caregivers (Physiotherapist, stretcher-bearer, Radiologic Technologist, Psychologist)
- Midwife
- Others

1. How long are you working in the same hospital service (years)

- <5
- ≥5

1. How long are you are you a graduate (years)

- <5
- ≥5

1. Do you have a history of professional burn-out or depression

- No
- Yes

1. What was your duty station during COVID-19 crisis (N=780)

- COVID Unit
- Non-Covid Unit
- Other professional activity
- Remote work

1. Did you manage COVID-19 patients

- Frequently
- Regularly
- Rarely
- Never

1. Have you been infected or did you have colleagues or relatives infected?

- Yes
- No

1. What is your marital situation (N=747)

- Single
- As a couple

1. What is your familial situation (N=471)

- No child
- One or several children

1. Has the COVID-19 crisis made you anxious?

- Yes
- No

1. What for were you anxious?

- Family
- Oneself
- In one’s work
- The others

1. Were you afraid of contaminating relatives?

- Yes
- No

1. Are you actually afraid of being infected?

- Yes
- No

1. Actually, do you feel good at work?

- Yes
- No

**Post-traumatic Stress Distress Checklist: PCL**

Instructions: Below is a list of problems that people sometimes have in response to a very stressful experience. Here we consider the Covid-19 crisis as a stressful event.

Please read each problem carefully and then circle one of the numbers to the right to indicate how much you have been bothered by that problem in the past month.

0 Not at all 1 A little bit 2 Moderately 3 Quite a bit 4 Extremely

1. Repeated, disturbing memories, thoughts, or images of the stressful experience?

2. Repeated, disturbing dreams of the stressful experience?

3. Suddenly acting or feeling as if the stressful experience were happening again (as if you were reliving it)?

4. Feeling very upset when something reminded you of the stressful experience?

5. Having physical reactions (e.g., heart pounding, trouble breathing, sweating) when something reminded you of the stressful experience?

6. Avoiding thinking about or talking about the stressful experience or avoiding having feelings related to it?

7. Avoiding activities or situations because they reminded you of the stressful experience?

8. Trouble remembering important parts of the stressful experience?

9. Loss of interest in activities that you used to enjoy?

10. Feeling distant or cut off from other people?

11. Feeling emotionally numb or being unable to have loving feelings for those close to you?

12. Feeling as if your future will somehow be cut short?

13. Trouble falling or staying asleep?

14. Feeling irritable or having angry outbursts?

15. Having difficulty concentrating?

16. Being "super-alert" or watchful or on guard?

17. Feeling jumpy or easily startled?


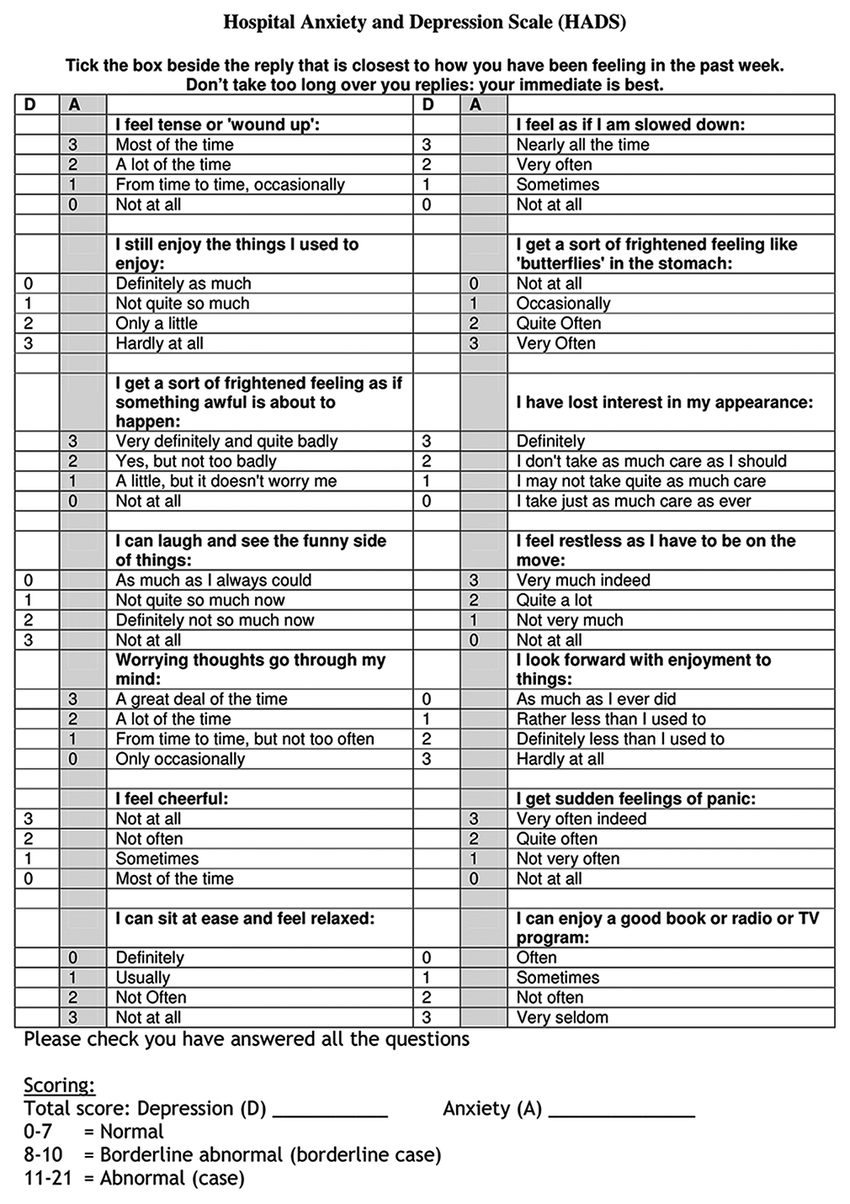

Supplement: Supplementary file 1 [file Table_1.DOCX]
